# Supplementary material for: A Humanized and Defucosylated Antibody against Podoplanin (humLpMab-23-f) Exerts Antitumor Activities in Human Lung Cancer and Glioblastoma Xenograft Models
Source: Cancers (Basel). 2023 Oct 20;15(20):5080. doi: 10.3390/cancers15205080 (PMC10605305; doi:10.3390/cancers15205080)
Supplement: Supplementary file 1 [file cancers-15-05080-s001.zip › cancers-2600525-supplementary.pdf]

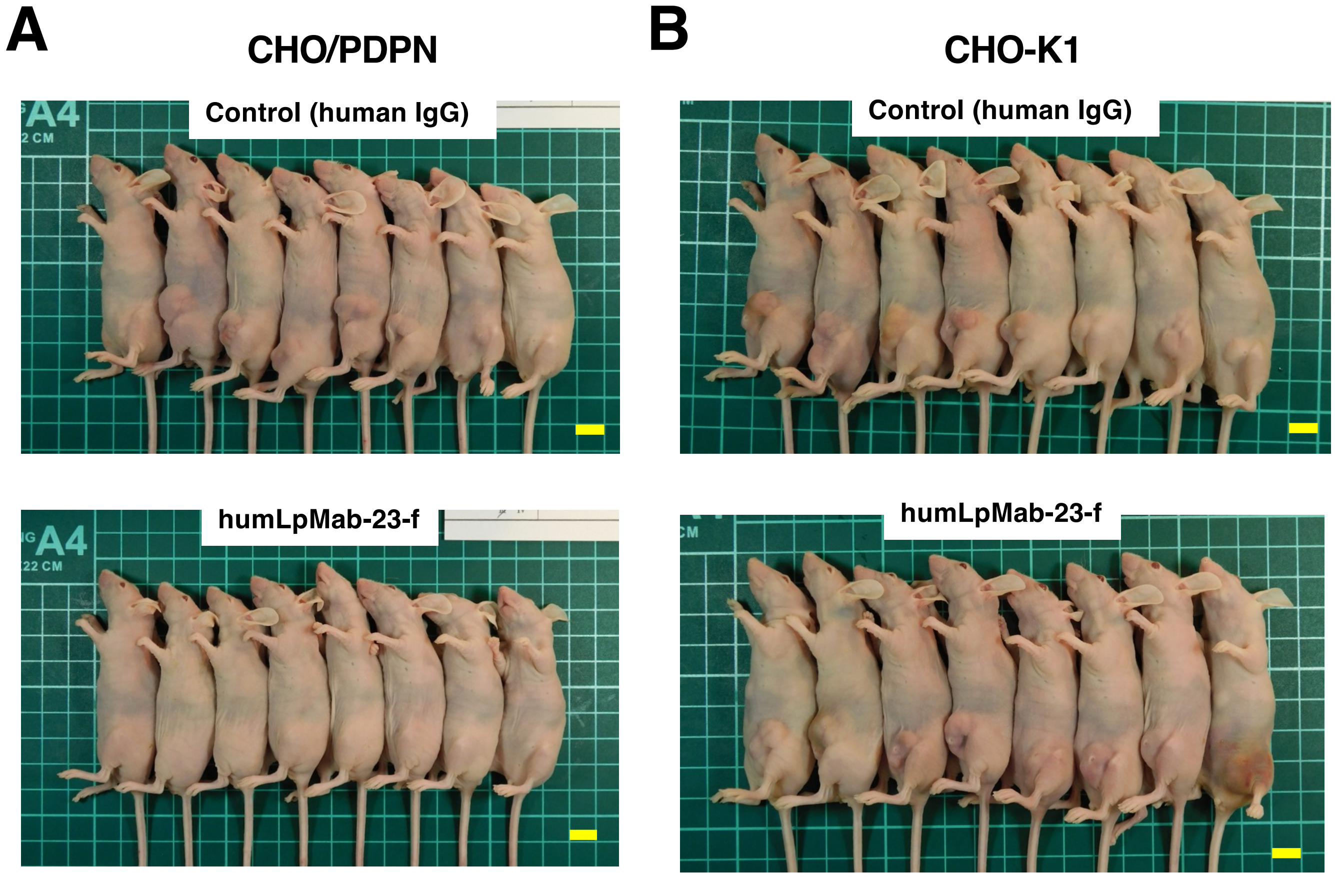

Supplementary Figure S1. Body appearance in CHO/PDPN (**A**) and CHO-K1 (**B**) xenografts-inoculated mice treated with human IgG and humLpMab-23-f on day 25. Scale bar, 1 cm.

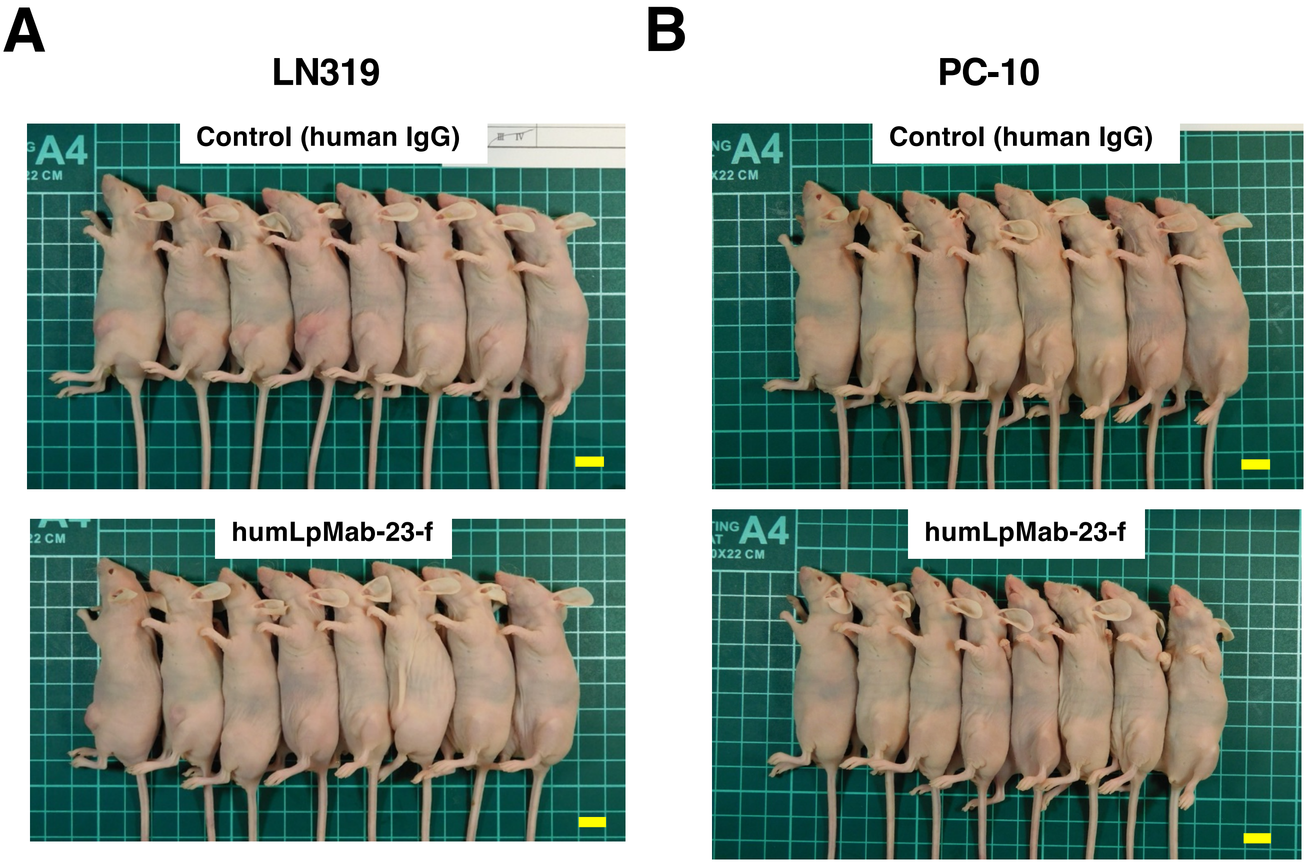

Supplementary Figure S2. Body appearance in LN319 (**A**) and PC-10 (**B**) xenografts-inoculated mice treated with human IgG and humLpMab-23-f on day 25. Scale bar, 1 cm.

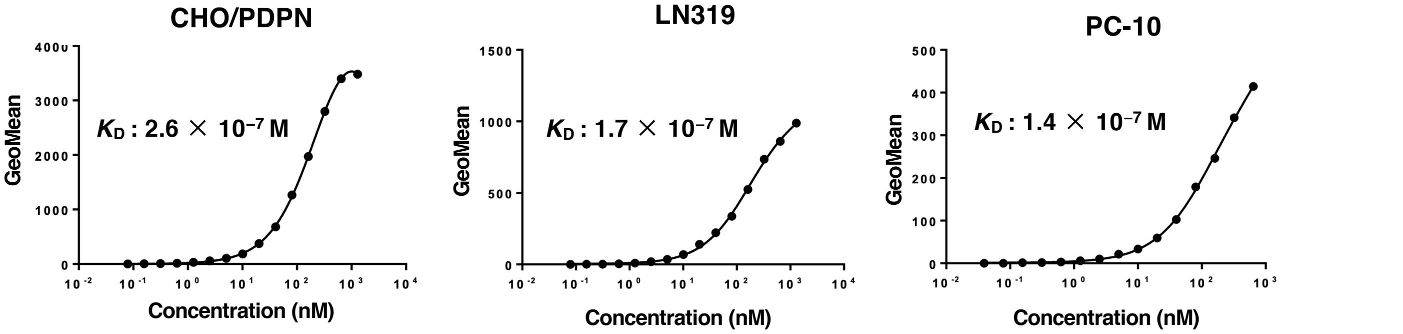

Supplementary Figure S3. Determination of the binding affinity of chLpMab-23 using flow cytometry. CHO/PDPN, LN319 and PC-10 cells were suspended in chLpMab-23 at indicated concentrations, followed by the addition of FITC-conjugated anti-human IgG. Fluorescence data were analyzed using the EC800 Cell Analyzer. The dissociation constant (K_D) was calculated by GraphPad Prism 8.
